# Immunohistochemical Evaluation of Aquaporin-4 and its Correlation with CD68, IBA-1, HIF-1α, GFAP, and CD15 Expressions in Fatal Traumatic Brain Injury

**DOI:** 10.3390/ijms19113544

**Published:** 2018-11-10

**Authors:** Margherita Neri, Alessandro Frati, Emanuela Turillazzi, Santina Cantatore, Luigi Cipolloni, Marco Di Paolo, Paola Frati, Raffaele La Russa, Aniello Maiese, Matteo Scopetti, Alessandro Santurro, Francesco Sessa, Rosanna Zamparese, Vittorio Fineschi

**Affiliations:** 1 Department of Morphology, Experimental Medicine and Surgery, University of Ferrara, Via Fossato di Mortara 70, 44121 Ferrara, Italy; margherita.neri@unife.it; 2 Department of Neurosciences, Mental Health, and Sensory Organs, Sant’Andrea Hospital, Sapienza University of Rome, Via di Grottarossa 1035, 00189 Rome, Italy; alessandro.frati@uniroma1.it; 3 IRCCS Neuromed, Via Atinense 18, 86077 Pozzilli, Italy; paola.frati@uniroma1.it (P.F.); raffaele.larussa@uniroma1.it (R.L.R.); 4 Institute of Legal Medicine, Department of Surgical Pathology, Medical, Molecular and Critical Area, University of Pisa, Ospedale Santa Chiara, Via Roma 55, 56126 Pisa, Italy; emanuela.turillazzi@unipi.it (E.T.); marco.dipaolo@unipi.it (M.D.P.); 5 Section of Legal Medicine, Department of Clinical and Experimental Medicine, University of Foggia, Ospedale Colonnello D’Avanzo, Via degli Aviatori 1, 71100 Foggia, Italy; santina.cantatore@unifg.it (S.C.); francesco.sessa@unifg.it (F.S.); 6 Department of Anatomical, Histological, Forensic and Orthopaedic Sciences, Sapienza University of Rome, Viale Regina Elena 336, 00185 Rome, Italy; luigi.cipolloni@uniroma1.it (L.C.); aniello.maiese@uniroma1.it (A.M.); matteo.scopetti@uniroma1.it (M.S.); alessandro.santurro@uniroma1.it (A.S.); 7 Unit of Legal Medicine, ASUR Area Vasta 5, Viale Marcello Federici, 63100 Ascoli Piceno, Italy; rosanna.zamparese@sanita.marche.it

**Keywords:** traumatic brain injury, aquaporin, genetic analysis, edema, computed tomography (CT) scan, hypoxia, microglia activation

## Abstract

Traumatic brain injury (TBI) is one of the leading causes of death and disability worldwide. Our understanding of its pathobiology has substantially increased. Following TBI, the following occur, edema formation, brain swelling, increased intracranial pressure, changes in cerebral blood flow, hypoxia, neuroinflammation, oxidative stress, excitotoxicity, and apoptosis. Experimental animal models have been developed. However, the difficulty in mimicking human TBI explains why few neuroprotective strategies, drawn up on the basis of experimental studies, have translated into improved therapeutic strategies for TBI patients. In this study, we retrospectively examined brain samples in 145 cases of death after different survival times following TBI, to investigate aquaporin-4 (AQP4) expression and correlation with hypoxia, and neuroinflammation in human TBI. Antibodies anti-glial fibrillary acid protein (GFAP), aquaporin-4 (AQP4), hypoxia induced factor-1α (HIF-1α), macrophage/phagocytic activation (CD68), ionized calcium-binding adapter molecule-1 (IBA-1), and neutrophils (CD15) were used. AQP4 showed a significant, progressive increase between the control group and groups 2 (one-day survival) and 3 (three-day survival). There were further increases in AQP4 immunopositivity in groups 4 (seven-day survival), 5 (14-dayssurvival), and 6 (30-day survival), suggesting an upregulation of AQP4 at 7 to 30 days compared to group 1. GFAP showed its highest expression in non-acute cases at the astrocytic level compared with the acute TBI group. Data emerging from the HIF-1α reaction showed a progressive, significant increase. Immunohistochemistry with IBA-1 revealed activated microglia starting three days after trauma and progressively increasing in the next 15 to 20 days after the initial trauma. CD68 expression demonstrated basal macrophage and phagocytic activation mostly around blood vessels. Starting from one to three days of survival after TBI, an increase in the number of CD68 cells was progressively observed; at 15 and 30 days of survival, CD68 showed the most abundant immunopositivity inside or around the areas of necrosis. These findings need to be developed further to gain insight into the mechanisms through which brain AQP4 is upregulated. This could be of the utmost clinicopathological importance.

## 1. Introduction

Current estimates from the World Health Organization (WHO) suggest that traumatic brain injury (TBI) will be the third leading cause of death and disability by the year 2020 [1,2]. Peeters et al. reported an average mortality rate of 10.53 per 10^5^ per year [3]. However, it is still difficult to calculate an average incidence and mortality rate since there is considerable variation in case definitions, including criteria and methods used in the studies [4].

Our understanding of the pathological mechanisms of TBI has substantially increased. However, many in-depth studies are needed, since the complex cascade of physiological and biochemical mechanisms in TBI are only partially understood.

The negative effects of brain trauma are determined not only by the entity of the primary injury resulting from a direct or indirect biomechanical force on the brain matter. Secondary events, such as edema formation, brain swelling, increased intracranial pressure, changes in cerebral blood flow associated with hypoxia, neuroinflammation, oxidative stress, excitotoxicity, and apoptosis [5,6], may occur with some delay, and may greatly affect the patient’s outcome [7]. Regardless of the cause of brain injury, brain edema almost always occurs and is heavily involved in the pathophysiology of brain damage following traumatic injury, being a central part of the vicious cycle of injury in TBI and the major determinant of patient prognosis [7,8,9,10,11,12].

Brain edema is caused by a synergy of molecular, cellular, structural, and functional changes in blood–brain barrier (BBB) function, microcirculation, and mediators, variously interacting in the different types of cerebral edema as first identified by Klatzo and subsequently modified [13,14]. In an animal model of TBI, Blixt et al. demonstrated that disintegration of the BBB was associated with increased permeability leading to an increased presence of immunoglobulin G (IgG) in the brain parenchyma at least four days post TBI [15].

Following TBI, brain edema occurs with vasogenic edema occurring rapidly after the injury, primarily in the center of the lesion, whereas cytotoxic edema has a later onset and is predominant [16,17]. There is growing evidence that members of aquaporins (small, hydrophobic membrane proteins regarded as a ‘pure water channel family’ that facilitate water transport) play an important role in traumatic brain edema [18,19,20,21,22].

Of these, aquaporin-4 (AQP4), the most abundant aquaporin in the brain [23], is the dominant contributor to the regulation of cerebral edema [24]. A biphasic role of AQP4 has been hypothesized: a negative role during edema formation and a positive role in the resolution phase [25]. Moreover, cross-talk between astrocyte activation, AQP expression, brain inflammation, and hypoxia exists in brain response to trauma [26,27] and this needs to be further clarified to gain a better understanding of the roles played by several mediators in the regulation of ion/water homeostasis in brain contusive injury. To achieve this depth of knowledge, it is of the utmost importance to consider that, in the brain, a complex network of neurons acts as a functional structure interacting with glial cells (i.e., astrocytes), cerebral blood vessels, and endothelial cells, and as part of a single physiological entity, the neurovascular unit (NVU) [28].

Experimental animal models of TBI have been developed, significantly advancing our understanding of the pathophysiology of TBI and, consequently, the development of many potential neuroprotective therapeutic strategies [29]. However, a deep translational gap still exists and few neuroprotective strategies have been translated into improved clinical care for patients with acute head trauma and other brain injuries [29]. One of the existing barriers is the lack of correspondence between traditional experimental models and the conditions experienced in real-life accidents [29]. Most human TBIs are due to blunt, nonpenetrating trauma, whereas most experimental animal models require fixation of the head in a stereotaxic frame or the creation of a cranial window [29]. In experimental animal models, a prolonged general anesthesia is used, which might potentially limit studies as anesthetic agents can influence the pathophysiology of secondary TBI [30]. Animal models closer to the reality of human TBI have been developed [29], minimizing the time of anesthesia, avoiding pre-injury surgery, and allowing the mouse head and body to be freely mobile during impact. These models showed a temporal course in the changes in AQP4 expression and localization after mild and moderate TBI. Several human studies have been conducted on traumatic patients exploring the cellular and molecular response of human brain tissue to traumatic injury. Fleming et al. explored the cellular inflammatory response in human spinal cords after traumatic injury [31], demonstrating an early activation of neutrophils and microglia, and subsequently generating a panel of oxidative and proteolytic enzymes that may cause secondary injury by enlarging the lesion and potentially worsening neurological dysfunction. The final conclusions provided an accurate view of the time course of the cellular inflammatory response after human spinal cord trauma, supporting the data that the critical time point for limiting neutrophil entry is within the first three days, ideally commencing within 12 h of injury. In surgical brain specimens from blunt traumatic patients, Suzuki et al. [32] demonstrated that vascular endothelial growth factors (VEGF), AQP1, and AQP4 were co-expressed in astrocytes located in the edematous tissue, confirming that astrocytes expressing AQPs and VEGF play a regulatory role in the water in- and out-flow in the injured tissue—a critical factor in the formation and resolution of brain edema. Another study performed on the contusioned cortex of human TBI focused on the degeneration and downregulation of astrocytes in traumatized brain leading to a reduction in the cellular expression of glutamate transporters, contributing to posttraumatic elevation of extracellular glutamate in humans [33]. A review study performed on brain surgical specimens from blunt traumatic patients provided evidence of the multifaceted role of astrocyte cells in severe traumatic human brain injuries [34,35,36,37,38,39].

In this study, we retrospectively examined brain samples in cases of death after different survival times following TBI to investigate AQP4 expression and correlation with neuroinflammation and hypoxia, to provide evidence of the complex role of AQP4 in human blunt TBI. To obtain data not related to genetic variants, the human *AQP4* gene (18q11.2–q12.1) was analyzed. On this basis, we investigated the presence of this single nucleotide polymorphism (SNP) on the *AQP4* gene in our study population, excluding all samples with this genetic variant.

## 2. Results

### 2.1. Genetic Analysis

**Figure 1:**
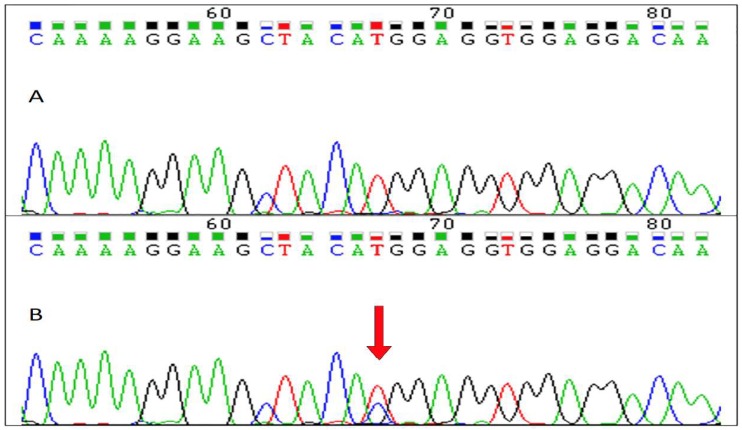
Aquaporin-4 (AQP4) DNA sequence in a wild type and a heterozygous individual. (**A**) DNA sequence electropherogram of a wild type individual showing wild type T at rs 3906956 position. (**B**) DNA sequence electropherogram showing the heterozygous rs 3906956 T > C in the individual with increased water permeability (red arrow).

In our cohort, we found 13 patients that were heterozygous for rs 3906956 T > C polymorphism in exon 4 of the *AQP4* gene (Figure 1). This variant was previously reported as the cause of increasing water permeability in relation to its location in the C-terminal domain. Therefore, we excluded these cases. As previously described, the C-terminal exon 4 includes phosphorylation sites (Ser276 and Tyr277) that could be associated with water permeability. In the Human Gene Mutation Database (www.hgmd.cf.ac.uk), only one naturally-occurring nonsynonymous single-nucleotide polymorphism (nsSNP) (rs 3906956, M278T) was previously reported in the *AQP4* gene, which could be potentially associated with changes in protein function, increasing water permeability [40]. We found 13 patients that were heterozygous for rs 3906956 T > C polymorphism in exon 4 of the *AQP4* gene. These patients were excluded because cases with edema could be related to inherent causes.

### 2.2. Evaluation of Brain Edema from CT Scans

**Figure 2:**
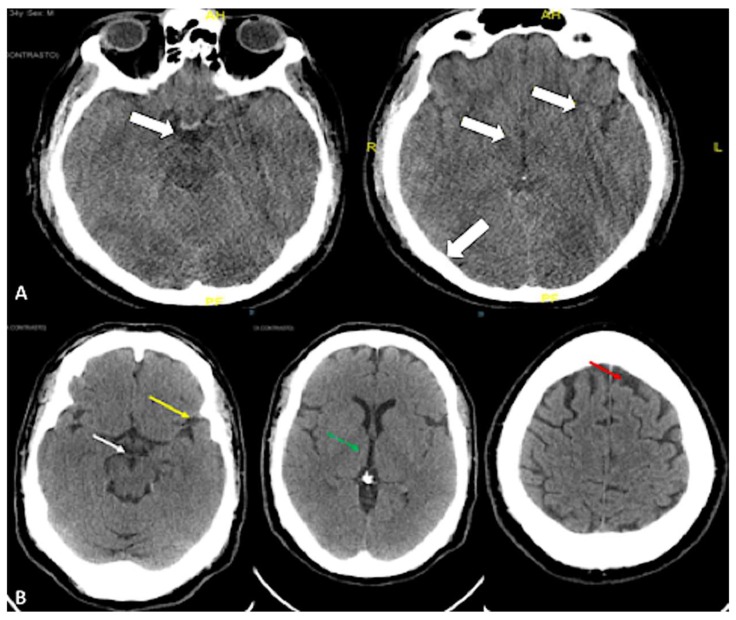
(**A**) The axial computed tomography (CT) scans evidence diffuse edema with cortical sulci, Sylvian fissure, third ventricle, and perimesencephalic cistern compressed but still visible. White arrow on the left scan shows the perimesencephalic cisterns, while white arrows on the right scan show the cortical sulci, third ventricle, and Sylvian fissure from left to right, respectively. (**B**) CT scan of a control case. The cortical sulci (red arrow), the perimesencephalic cisterns (white arrow), the Sylvian fissure (yellow arrow), and third ventricle (green arrow with dotted line) are clearly visible. No radiological sign of edema is evidenced.

**Figure 3:**
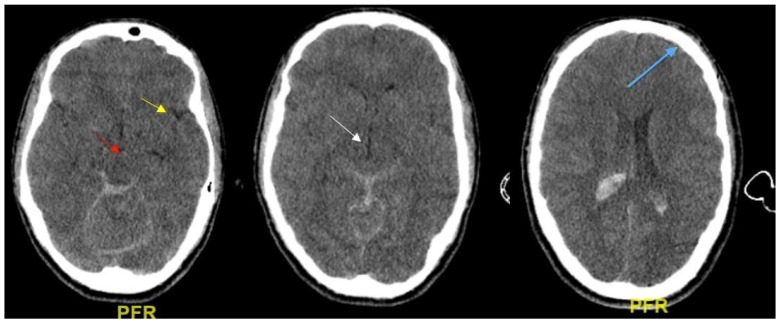
The axial CT scans evidence diffuse edema with subarachnoid and intracerebral hemorrhage: cortical sulci (blue arrow), and perimesencephalic cisterns (red arrow) are not visible, while Sylvian fissure (yellow arrow) and third ventricle (white arrow) are compressed but still visible.

**Figure 4:**
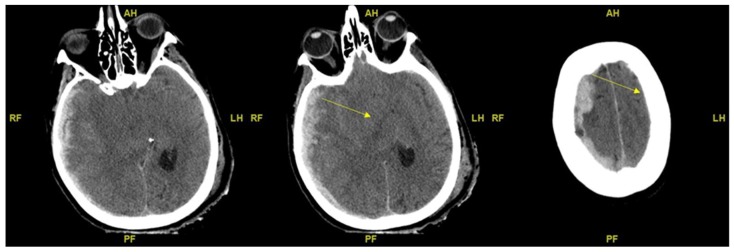
The axial CT scans evidence diffuse brain edema and swelling, with severe midline shift due to multiple brain contusions and acute subdural and subarachnoid hemorrhage. Cortical sulci, Sylvian fissure, third ventricle, and perimesencephalic cistern are either compressed or not visible. From left to right side yellow arrows show the third ventricle compressed and dislocated and the cortical sulci compressed and pushed toward the skull.

Based on the available CT scans, edema/brain water burden was assessed semiquantitatively on a scale of 0 to 3, as a modification of Ito et al.: −: no detectable signs of edema; +/−: cortical sulci, Sylvian fissure, and third ventricle and perimesencephalic cistern compressed, but visible, in CT scans (Figure 2); ++: one or two of the above structures not visible (Figure 3); and +++: three or four of these structures not visible (Figure 2, Figure 3 and Figure 4).

**Figure 5:**
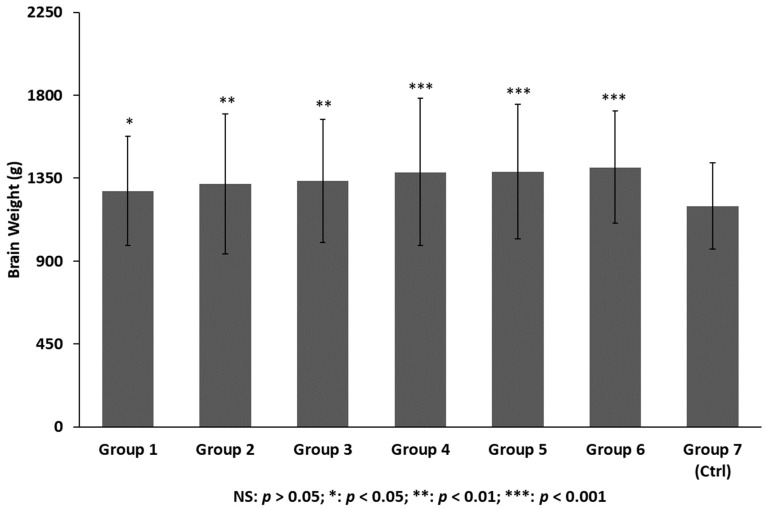
Brain weight: the differences among multiple groups were assessed using one-way analysis of variance (ANOVA) with a significance level at *p* < 0.05. Post-hoc comparison between groups was further detected using the least significant difference (LSD) method.

The weight of the brains was carefully evaluated and estimated in the TBI groups and control group (Figure 5). Table 1 summarizes the results.

### 2.3. Histological Examination

In group 1 (immediate death), histological examination of the contusion areas showed evidence of a hemorrhagic core with abundant necrotic tissue. The areas of necrosis became surrounded by inflammatory cells, such as neutrophils and macrophages, starting from one day of survival after injury (group 2). In the control group and group 1, neutrophils were all intravascular. They became increasingly evident around the margin of the necrotic areas; group 4 (seven day survival) showed the most abundant macrophages inside or around the areas of necrosis. Weeks after TBI (groups 5 and 6), only a few scattered neutrophils were observed through the reparative or cystic areas.

Histological features of brain edema in the pericontusion areas became more evident in groups 2 and 3 where swelling of the astrocytes and the neuronal dendrites was present (group 2) followed by the appearance of swollen astrocytes and shrinkage of the neurons that become eosinophilic, with pyknosis of the nuclei (group 3). Vacuolated spaces around blood vessels were also present in both groups. Similar histological findings were observed in more distant areas and in the contralateral tissue samples. In groups 4, 5, and 6 (7, 14, and 30 days’ survival, respectively) the swelling of astrocytes and neurons remained evident for all regions assessed. An increasing proliferation of reactive astrocytes in the tissue proximal to the contusion was detected; at high magnification and on confocal three-dimensional analysis, astrocytes became visibly larger with increased soma sizes and thickened processes.

### 2.4. Immunohistochemistry

The immunohistochemical study of brain areas samples from the different groups produced the results as shown in Table 2.

**Figure 6:**
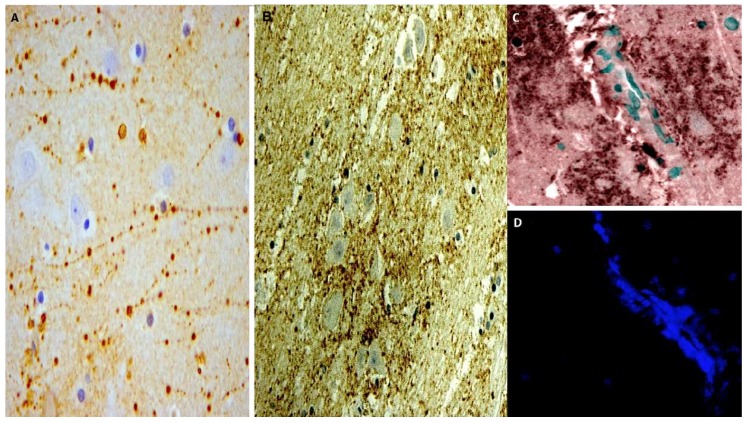
(**A**) AQP4 expression in group 5 (14 days): widespread immunopositivity is evidenced in brown color. (**B**) Diffuse perivascular AQP4 positivity reactions (brown) in group 6 (30 days). (**C**) Double immunoreactions for CD15 and AQP4: isolated immunopositivity in group 5 (14 days) for CD15 demonstrated by the dark reaction, and diffuse immunopositivity for AQP4 in green. (**D**) Confocal microscopy: diffuse reaction in group 6 (30 days); AQP4 is intensely positive (blue color) (magnification 150×).

Immunohistochemistry was used to determine whether the expression of AQP4 changed at different time points of survival. An injury-induced increase in AQP4 immunopositivity was observed (control group versus all trauma groups *p* < 0.01, except for group 1, almost immediate death). When comparing the different groups, a significant (*p* < 0.001) increase in AQP4 immunostaining was detected between group 2 (one day survival) and 3 (three day survival), and between group 2 and 4 (seven day survival), thus suggesting an upregulation of AQP4 at three to seven days compared to one day. There were increases in AQP4 immunopositivity in group 5 (14 days survival), and the *p*-value was 0.0016. A significant increase was seen between groups 2, 3, and 4 and groups 5 and 6 (30 day survival), thus suggesting an upregulation of AQP4 at 15 to 30 days compared to one day (Figure 6A–D).

**Figure 7:**
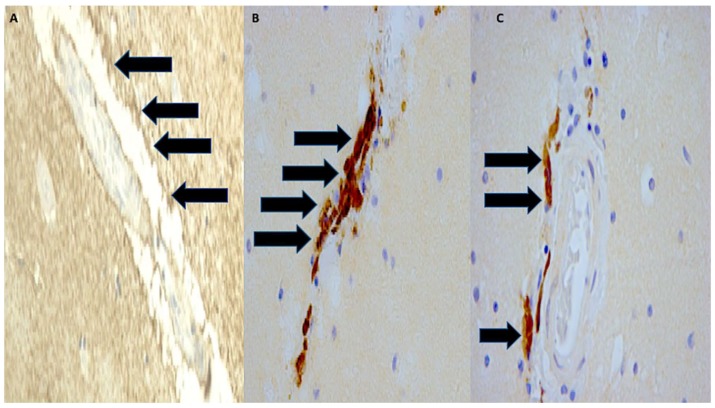
HIF-1α immunopositivity (black arrows) increased significantly at (**A**) early time points of survival (one and three days of survival) until seven days. (**B**,**C**) Group 4: diffuse perivascular positivity) (magnification 150×).

HIF-1α immunoexpression increased significantly at early time points of survival (one and three’ days survival, group 2 and 3) and in group 4 compared to control cases (group 7) and immediate deaths (group 1). Lesser statistical significance was seen when group 1 was compared with groups 5 and 6 (Figure 7A–C).

**Figure 8:**
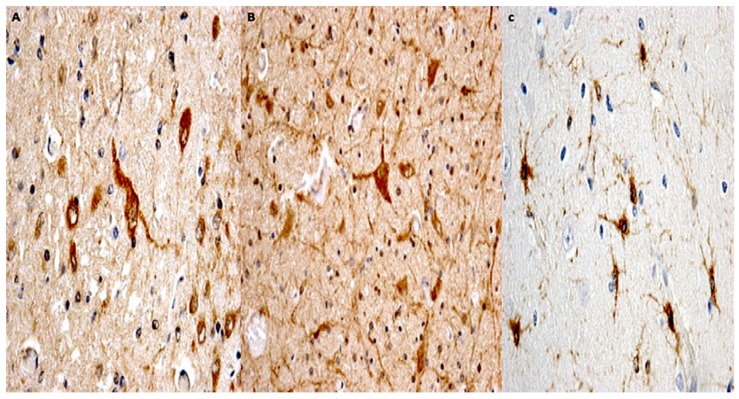
(**A**) A significant increase in GFAP immunopositivity (brown reactions) was observed in group 4: swollen astrocytes and neurons remained evident for all the regions assessed (magnification 200×). (**B**,**C**) Group 6: an increasing in reactive astrocytes was detected (magnification 150×); at high magnification astrocytes became visibly larger with increased soma sizes and thickened processes (magnification 200×).

A significant (*p* < 0.05) increase in anti-GFAP immunopositivity was observed in all groups compared to group 1 and the control group, where a basal, perivascular GFAP immunopositivity was observed. Statistical analysis showed significant post-injury time effects on GFAP immunoexpression between groups 2, 3, and 4 (*p* < 0.001), suggesting an upregulation of GFAP expression at 72 h compared to 24 h. Significant increases in GFAP immunopositivity (*p* < 0.001) were observed in groups 5 and 6 (14 and 30 days following TBI) when compared to groups 1 and 2. The corresponding histological observation showed that GFAP staining darkened and astrocytes became larger and entangled; at high magnification, astrocytes became visibly larger with increased soma sizes and thickened processes (Figure 8A–C).

**Figure 9:**
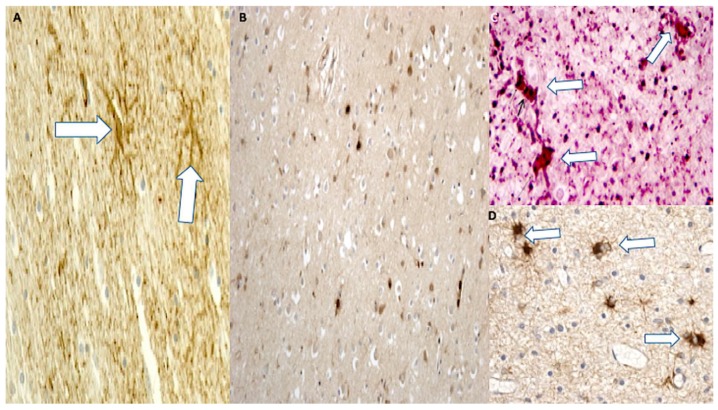
(**A**) CD68 immunopositivity (white arrows) increased significantly at early time points of survival (one and three day survival) until 30 days (magnification 150×). (**B**) Group 6: diffuse brown positivity (magnification 100×). (**C**) IBA-1 reactions showing dendritic morphology (white arrows) (magnification 150×). (**D**) An increase in the number of IBA-1 immunopositive cells (white arrows) was progressively observed at high magnification (200×) (group 6).

In control cases and in group 1, a basal macrophage/phagocytic activation immunopositivity was observed mostly around blood vessels. Starting from one to three days of survival after TBI, an increase in the number of CD68 cells was progressively observed in conjunction with a change in their morphology. CD68+ cells became larger with shorter and thicker processes (Figure 9A). Groups 5 and 6 (15 and 30 days’ survival) showed the most abundant CD68 immunopositivity inside or around the areas of necrosis (Figure 9B).

Immunopositivity to IBA-1 was observed in control cases and in group 1, with cells showing dendritic morphology (Figure 9C). Starting from three days of survival after TBI, an increase in the number of cells was progressively observed, which then stabilized in the further survival period (groups 4, 5, and 6), in conjunction with the CD68+ cells, which progressively showed the most abundant immunopositivity (Figure 9D).

**Figure 10:**
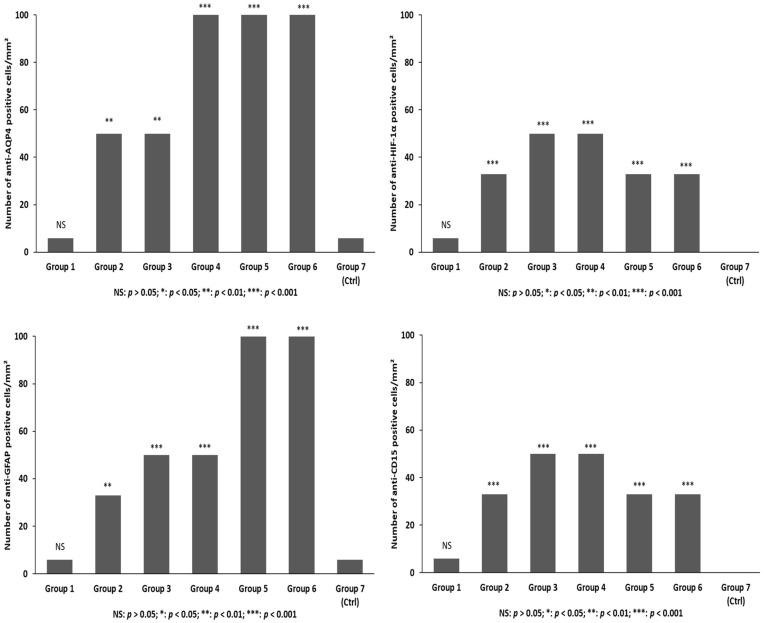
The differences among multiple groups were assessed using one-way ANOVA with a significance level at *p* < 0.05. Post-hoc comparison between groups was further detected using the least significant difference (LSD) method.

The areas of necrosis became surrounded by inflammatory cells such as neutrophils labeled by their expression of CD15 starting from one day of survival after injury (group 2). In the control group and in group 1, neutrophils were all intravascular. They became increasingly evident around the margin of the necrotic areas. Weeks after TBI (groups 5 and 6), only a few scattered neutrophils were observed through the reparative or cystic areas (Figure 10).

### 2.5. Statistical Analysis

**Figure 11:**
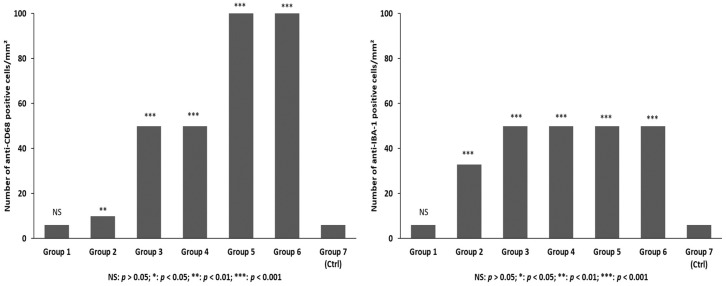
CD68 and IBA-1 immunopositivity. The differences between multiple groups were assessed using a one-way ANOVA with a significance level at *p* < 0.05. Post-hoc comparison between groups was further detected using the LSD method.

Statistical analysis of the immunohistochemical findings and gradation of the immunohistochemical reaction in the brain samples for each group is summarized in Figure 10 and Figure 11.

## 3. Discussion

In this study, we investigated the expression of AQP4, the primary water channel in the brain, in fatal human traumatic brain injury at several evolution phases. The pathophysiology of traumatic brain injury is complex. We analyzed the correlation between clinical and computed tomography (CT) data with histological results to demonstrate the mechanisms by which brain AQP4 is upregulated. It is thought that metabolic dysfunction, excitotoxicity, oxidative stress, edema formation, inflammation, and cell death (necrosis and apoptosis, respectively) are involved [6,7]. Difficulty in the use of inflammatory markers, such as immunohistochemical markers of TBI in the early stage, is due to an overlap of secondary changes after traumatic event (e.g., hypoxia and edema) that can lead to difficulty and errors in estimating survival time.

AQP4 shows a significant and progressive increase between control group and groups 2 (one day survival) and 3 (three day survival), from the acute stages of the traumatic insult. There were further increases in AQP4 immunopositivity in groups 4 (seven day survival), 5 (14 day survival), and 6 (30 day survival), suggesting an upregulation of AQP4 at 7 to 30 days compared to one day.

GFAP, characterized by later expression, showed its highest expression in group 5 (14 day survival) and 6 (30 day survival) at the astrocytic level, compared with groups 1–3 and group 4 (seven day survival). Astrocytic cell bodies enlarged and the astrocytic end-feet were thicker and prominent.

Data emerging from the HIF-1α reaction agreed with those reported in the literature, showing a progressive, significant increase until group 4 (seven day survival). In hypoxia, upregulation of HIF-1α, associated with disruption of BBB and increased permeability of blood vessels, has been linked to an upregulation of AQP4, which involves astrocytes in brain edema [41].

As defined by Hoogland et al., microglial cells are defined as activated based on the following criteria. (1) Microglia showed an activated morphology based on immunohistochemical staining, (2) there was a significant increase in number and/or size of microglia compared to the control group, and (3) there was a significant increase in expression of a microglial marker [42,43].

Our immunohistochemistry study with IBA-1 revealed that microglia were activated starting three days after trauma and progressively increasing for the next 15 to 20 days after the initial trauma. CD68 expression demonstrated a basal macrophage/phagocytic activation mostly around blood vessels. Starting from one to three days of survival after TBI, an increase in the number of CD68 cells was progressively observed in conjunction with a change in their morphology: cells became larger with shorter and thicker processes. At 15 and 30 days of survival, CD68 showed the most abundant immunopositivity inside or around the areas of necrosis. At all time points microglia were morphologically ramified with fine processes.

The areas of necrosis became surrounded by neutrophils (CD15) starting from one day of survival after injury. They became increasingly evident around the margin of the necrotic areas (group 4, seven day survival); only a few scattered neutrophils were observed through the reparative or cystic areas weeks after TBI. The progressive increase in AQP4 we observed in the present retrospective study, as opposed to most other studies, may be interpreted as variations in the temporal course of injury following trauma. Other studies came to different conclusions. As described by Blixt [15], in cytotoxic edema induced by ischemia, the amount of edema is reduced in AQP4 deficient animals, water accumulation is intracellular, and astrocytes, normally diffusely populated by AQP4 channels, would be protected from swelling in knockout models. Conversely, vasogenic edema increased in AQP4 deficiency created by hydrostatic forces for a disrupted BBB; the explanation is that edema is mainly extracellular and was conjectured to be cleared by fluid elimination through AQP4 water channels located in the glial linings of the ventricles [15].

AQP water channels may have a pivotal role in maintaining water homeostasis in the brain [44], and these channels have been demonstrated to be involved in the development and resolution of cytotoxic and vasogenic edema [25,45,46]. AQP4 shows polarized distribution, being mainly expressed in astrocyte foot processes surrounding capillaries; astrocyte processes, which comprise the glial limiting membrane; and in ependymal cells, with microvilli expressing no AQP4 at all [47,48,49]. In many pathological conditions, a redistribution of AQP4 occurs in membrane domains apart from end-feet areas, due to the degradation of the proteoglycan agrin by the matrix metalloproteinase 3 [29,50,51,52]. AQP4 is abundant at sites of fluid transport, including pial and ependymal surfaces in contact with cerebrospinal fluid (CSF), subarachnoid space, and the ventricular system [48,53]. This distribution indicates that AQP4 is crucial in maintaining water balance in the brain with a versatile function, facilitating water fluxes into and out of the brain parenchyma [54]. Edema can be viewed as a dysfunction of aquaporins to properly prevent or facilitate water movement [25].

An overexpression of AQP4 has been demonstrated in the reactive astrocytes after cerebral injury [55]. AQP4 acts as bidirectional water transport, which enables it to contribute to both edema formation at early time points, and the clearance of water from the brain into blood vessels at later time points [56]. AQP4 is critical both in the movement of water into swelling astrocytes [45,57], as seen in cellular cytotoxic edema, as well as in the resolution of vasogenic edema [47] after brain injury.

Pinchi et al. indicated that miR-21, miR-92, and miR-16 have a high predictive power in discriminating trauma brain cases from controls and could be promising biomarkers as a strong predictor of survival, and useful tools for post-mortem diagnosis of traumatic brain injury [58,59]. The presence of AQP4 seems to play an acute detrimental role, but at a later phase starting from around seven days post injury and for at least one month. AQP4 may play a beneficial role that seems to be related to the inhibition of microglia and promotion of edema resolution [60,61,62,63].

## 4. Materials and Methods

### 4.1. Study Population

The clinical data and the autopsy records of the autopsies performed in the Department of Forensic Pathology of the Universities of Rome and Foggia, Italy, over the period of 1999 to 2015 were evaluated retrospectively. We identified 388 cases in which contusive, nonpenetrating traumatic brain injury was revealed as cause of death. Since age-related differences have been reported in post-traumatic brain swelling and vascular changes after TBI [28,36,37,38], we excluded all TBI cases in infants and children. In our selected cases, the age of the deceased ranged between 24 and 76 years. Only cases with negative toxicology for drug and ethanol abuse were included in the study. For our study, we excluded 13 patients with genetic polymorphism rs 3906956. Following these criteria, 145 cases were selected for our retrospective analysis. In the final cohort, a total of 91 men and 54 women were included, as reported in Table 3. We divided the study population into six groups, each consisting of a similar number of cases, according to the survival interval after TBI. The control group (group 7) included 25 cases, selected among sudden, nontraumatic deaths, with negative toxicological analysis, in which no evidence of TBI was detected at post-mortem examination. The age of the control group ranged between 20 and 71 years; 18 men and 7 women were included.

The processing of the data reported in this paper was covered by the general authorization to process personal data for scientific research purposes granted by the Italian Data Protection Authority (1 March 2012 as published in Italy’s Official Journal no. 72 dated 26 March 2012), since the data do not entail any significant personalized impact on data subjects.

Our study did not involve the application of experimental protocols; therefore, it did not require approval by an institutional and/or licensing committee. In all cases, local prosecutors opened an investigation, ordering that an autopsy be performed to clarify the exact cause of death.

This investigation conforms to the principles outlined in the declaration of Helsinki.

### 4.2. Genetic Analysis

For each group, 10 sample blocks, sections of 10 µm were cut from each specimen with a new sterile blade and outer sections were discarded. Four or five scrolls from serial sections were placed in each tube (Eppendorf AG, Hamburg, Germany) for DNA extraction protocols.

#### 4.2.1. DNA Extraction Protocols

We extracted the samples with QIAamp DNA Formalin-Fixed Paraffin-Embedded (FFPE) Tissue Kit (Qiagen, Valencia, CA, USA), according to the manufacturer’s instructions with this modification: the pellet was digested with a tissue lysis (ATL) buffer (Qiagen, Valencia, CA, USA) and proteinase K overnight.

#### 4.2.2. PCR Amplification and DNA Sequencing

We built primers for M278T SNP, allele A/G, rs 3906956, with Primer3web version 4.0.0 (ELIXIR, Tartu, Estonia). All primers were synthesized (Eurofins Genomics, Ebersberg, Bayern, Germany): AQPEX4shortFw 5′ ≥ 3′ AAAGAAGCCTTCAGCAAAGC; AQPEX4shortRw 5′ ≥ 3′ GGTCAACGTCAATCACATGC. Polymerase chain reaction (PCR) was carried out using 50-µL volume samples in a PCR thermocycler MasterCycler (Eppendorf AG, Hamburg, Germany). The PCR conditions for the 35 cycles were denaturation 95 °C for 30 s, annealing 55 °C for 30 s, and extension 72 °C for 1 min. Each sample contained 0.1 mg genomic DNA, 10 pmol of each primer, 125 mM deoxyribonucleoside triphosphate, 5 mM tris (hydroxymethyl) aminomethane (Tris) HCl (pH 8.3), 50 mM KCl, 1.5 mM MgCl_2_, and 1U Taq polymerase (Applied Biosystems, Foster City, CA, USA). Amplified DNA fragments were subjected to direct cycle sequence analysis using the Taq dye-deoxy terminator method with reverse primer (Applied Biosystems, Foster City, CA, USA) and an ABI PRISM 3100 Genetic Analyzer sequencer (Applied Biosystems, Foster City, CA, USA).

### 4.3. Evaluation of Edema CT Scan

Based on the available CT scans, edema/brain water burden was assessed semiquantitatively on a scale of 0–3, modified from Ito et al.: −: no detectable signs of edema; +/−: cortical sulci, Sylvian fissure, third ventricle and perimesencephalic cistern compressed, but visible, in CT scans; ++: one or two of the above structures not visible; and +++: three or four of these structures not visible. The weight of the brains had been carefully evaluated and estimated in the TBI groups and control group.

### 4.4. Histological and Immunohistochemical Analysis

We retrospectively examined brain samples taken at autopsy. Brain tissues from cases and controls were fixed in 10% buffered formalin for 48 h. Then 4 µm-thick paraffin-embedded sections were cut and stained with hematoxylin and eosin (H&E) stain. The areas of intense tissue injury, either in the form of necrotic tissue (in the shorter survival cases) or as cystic change (in the longer surviving cases), were excluded from immunohistochemical investigation. In addition, immunohistochemical investigation of the grey matter of the frontal and temporal sections area was performed utilizing antibodies anti-glial fibrillary acid protein (GFAP) (Agilent Dako, Copenhagen, Denmark), aquaporin-4 (AQP4) (Santa Cruz Biotechnology, Santa Cruz, CA, USA), hypoxia induced factor-1α (HIF-1α) (Santa Cruz Biotechnology Santa Cruz, CA, USA), macrophage/phagocytic activation (CD68) (Santa Cruz Biotechnology Santa Cruz, CA, USA), ionized calcium-binding adapter molecule-1 (IBA-1) (Abcam, San Francisco, CA, USA), and neutrophils CD15 (Agilent Dako, Copenhagen, Denmark). We used 4-µm-thick paraffin sections mounted on slides covered with (3-aminopropyl)triethoxysilane (Fluka, Buchs, Switzerland). Pretreatment was necessary to facilitate antigen retrieval and to increase membrane permeability to antibodies anti-AQP4, CD 15, and HIF-1α boiling in 0.25 M ethylene-diamine-tetraacetic acid (EDTA) buffer, to antibodies anti-macrophage/phagocytic activation (CD68) and ionized calcium-binding adapter molecule-1 (IBA-1) for 15 min in proteolytic enzyme (Agilent Dako, Copenhagen, Denmark), at 20 °C. No pretreatment was necessary for antibody anti-GFAP. The dilution of primary antibody was: CD15, 1:50 ratio; HIF-1α, 1:100 ratio; CD68 and IBA-1, 1:200 ratio; GFAP, 1:300 ratio; and AQP4, 1:500 ratio. The primary antibody was incubated for 120 min at 20 °C. The detection system utilized was the labeled streptavidin-biotin (LSAB+) Kit (Agilent Dako, Copenhagen, Denmark), a refined avidin-biotin technique in which a biotinylated secondary antibody reacts with several peroxidase conjugated streptavidin molecules.

In addition, double antigen labeling, using antibody anti-AQP4, anti CD15, anti CD68, anti IBA-1 and anti GFAP, was performed.

The samples were examined under a confocal microscope True Confocal Scanner (Leica TCS SPE, Milan, Italy), and a three-dimensional reconstruction was performed. A preliminary semiquantitative evaluation of the immunohistochemical findings was performed by two different investigators without prior knowledge. All measurements were taken using the same magnification of image (10×) by the same two examiners. A third blind microscopic evaluator was involved to weigh the histological evidence.

Statistical analysis of the immunohistochemical findings and gradation of the immunohistochemical reactions was performed as follows. Responses about antibody anti-AQP4, anti-GFAP, anti-HIF-1α, anti-CD68, anti-IBA-1, and anti-CD15 expressions in brain specimens were evaluated, where NS (not significant): *p* > 0.05; * *p* < 0.05; ** *p* < 0.01; and *** *p* < 0.001. Intensity of immunopositivity was assessed semiquantitatively on a 0–4 scale as follows, −: no immunoreactivity (0%); +: mild immunopositivity in scattered cells (10%); ++: immunopositivity in up to one third of cells (33%); +++: immunopositivity in up to two- third of cells (70%); and ++++: strong immunopositivity in the majority or all cells (100%). In cases of divergent scoring, a third observer decided the final category.

### 4.5. Statistical Analysis

The statistical analysis was assigned a numerical value from 0 to 5 for each single sample of each group, according to the score attributed to the semi-quantitative evaluation. All the data was presented as mean ±SE. Statistical analysis was performed with SPSS for Windows version 13.0 (SPSS Inc., Chicago, IL, USA). The differences between multiple groups were assessed using a one-way analysis of variance (ANOVA) with a significance level at *p* < 0.05. Post-hoc comparison between groups was detected using the least significant difference (LSD) method.

## 5. Conclusions

Cerebral edema with brain swelling remains the most significant predictor of outcome in TBI. Edema formation leads to an expansion of brain volume as it increases intracranial pressure, impairs cerebral perfusion and oxygenation, and contributes to ischemic injuries and inflammatory response.

These findings need to be further examined to provide insight into the mechanisms by which brain AQP4 is upregulated. This could be of utmost clinicopathological importance.

## Figures and Tables

**Table 1:** Brain weight and computed tomography (CT) determination of edema/brain water burden.

Group	Number of Cases	GCS Evaluation	Time of Death After Trauma	Edema/Brain Water Burden	Brain Weight
1	25	Not evaluated	Almost immediate	+/−	1280 ± 296 g
2	24	4–8	1 day (20 ± 6 h)	++	1320 ± 380 g
3	24	3–8	3 days (72 ± 10 h)	++	1336 ± 334 g
4	24	3–8	7 days (6 ± 2 days)	+++	1384 ± 400 g
5	24	3–8	14 days (14 ± 4 days)	+++	1386 ± 364 g
6	24	3–8	30 days (30 ± 10 days)	+++	1410 ± 304 g
7 (Control)	25	Not evaluated	Not determined	+/−	1200 ± 234 g

Based on the available CT scans, edema/brain water burden was assessed semiquantitatively on a scale of 0–3, as a modification of Ito et al. as follows: − no detectable signs of edema; +/− cortical sulci, Sylvian fissure, third ventricle, and perimesencephalic cistern compressed, but visible, in CT scans; ++ one or two of the above structures not visible; +++ three or four of these structures not visible [39]. GCS—Glasgow Coma Scale.

**Table 2:** Semiquantitative evaluation and statistical analysis of the immunohistochemical findings and gradation of the immunohistochemical reaction in the brain samples.

Antibody	Group 1	Group 2	Group 3	Group 4	Group 5	Group 6	Group 7 (Control)
AQP4	+/−	+++	+++	++++	++++	++++	+/−
HIF-1α	+/−	++	+++	+++	++	++	−
GFAP	+/−	++	+++	+++	++++	++++	+/−
CD68	+/−	+	+++	+++	++++	++++	+/−
IBA-1	+/−	++	+++	+++	+++	+++	+/−
CD15	+/−	++	+++	+++	++	++	−

Analysis of the immunohistochemical findings and gradation of the immunohistochemical reactions. Responses for antibodies anti-aquaporin-4 (AQP4), anti-hypoxia induced factor-1α (HIF-1α), anti-glial fibrillary acid protein (GFAP), anti-macrophages (CD68), anti-ionized calcium-binding adapter molecule-1 (IBA-1), and anti-neutrophils (CD15) expressions in brain specimens. Intensity of immunopositivity was assessed semiquantitatively in the scale of 0 to 4 as follows, −: no immunoreactivity (0%); +: mild immunopositivity in scattered cells (10%); ++: immunopositivity in up to one third of cells (33%); +++: immunopositivity in up to two-third of cells (70%); and ++++: strong immunopositivity in the majority or all cells (100%). In cases of divergent scoring, a third observer decided the final category.

**Table 3:** Study population divided according to the survival interval after traumatic brain injury (TBI).

Group	Number of Cases	Sex	Time of Death after Trauma
1	25	16 men; 9 women	Almost immediate
2	24	15 men; 9 women	1 day (20 ± 6 h)
3	24	16 men; 8 women	3 days (72 ± 10 h)
4	24	17 men; 7 women	7 days (6 ± 2 days)
5	24	12 men; 12 women	14 days (14 ± 4 days)
6	24	15 men; 9 women	30 days (30 ± 10 days)
7 (Control)	25	18 men; 7 women	Undetermined
